# Use of FDG-PET in differentiating benign from malignant compression fractures

**DOI:** 10.1007/s00256-008-0452-5

**Published:** 2008-05-01

**Authors:** Miriam A. Bredella, Brendan Essary, Martin Torriani, Hugue A. Ouellette, William E. Palmer

**Affiliations:** Department of Radiology, Massachusetts General Hospital, Yawkey 6E, 55 Fruit Street, Boston, MA 02114 USA

**Keywords:** Spine, Compression fractures, Malignant, Benign, FDG-PET

## Abstract

**Objective:**

The objective was to evaluate the use of fluorodeoxyglucose positron emission tomography (FDG-PET) in differentiating benign from malignant compression fractures.

**Patients and methods:**

In a retrospective analysis, we identified 33 patients with 43 compression fractures who underwent FDG-PET. On FDG-PET the uptake pattern was recorded qualitatively and semiquantitatively and fractures were categorized as benign or malignant. Standardized uptake values (SUV) were obtained. MRI, CT, and biopsy results as well as clinical follow-up for 1–3 years served as standards of reference. The Student’s *t* test was used to determine whether there was a statistically significant difference between the SUV for benign and malignant compression fractures.

**Results:**

There were 14 malignant and 29 benign compression fractures, including 5 acute benign fractures. On FDG-PET, 5 benign fractures were falsely classified as malignant (false-positive). Three of these patients underwent prior treatment with bone marrow-stimulating agents. There were two false-negative results. Sensitivity, specificity, positive predictive value, negative predictive value, and accuracy of FDG-PET in differentiating benign from malignant compression fractures were 86%, 83%, 84%, 71%, and 92% respectively. The difference between SUV values of benign and malignant fractures was statistically significant (1.9 ± 0.97 for benign and 3.9 ± 1.52 for malignant fractures,* p* < 0.001). SUV of benign acute and chronic fractures were not statistically significant.

**Conclusion:**

Fluorodeoxyglucose positron emission tomography is useful in differentiating benign from malignant compression fractures. Therapy with bone marrow-stimulating agents can mimic malignant involvement.

## Introduction

Differentiating between malignant and benign vertebral compression fractures can represent a diagnostic challenge and is particularly difficult in elderly patients, who frequently have a history of malignancy and are also predisposed to benign compression fractures from osteoporosis or treatment-related changes [1–3]. Differentiating between benign and malignant compression fractures has important therapeutic and prognostic implications.

Computed tomography (CT) and magnetic resonance imaging (MRI) are routinely used in the evaluation of compression fractures; however, these imaging modalities do not always permit definite diagnosis [2–5]. In contrast to MRI or CT, fluorodeoxyglucose positron emission tomography (FDG-PET) can yield metabolic information that is based on increased glucose metabolism of malignant and inflammatory lesions. Tumor cells typically accumulate FDG, while traumatic fractures are not expected to significantly accumulate FDG. Therefore, FDG-PET may allow differentiation between malignant and benign compression fractures [6–9].

The purpose of our study was to evaluate the use of FDG-PET in differentiating benign from malignant compression fractures.

## Materials and methods

This study was approved by the institutional review board of our institution, which waived the need for informed consent. The study was compliant with the Health Insurance Portability and Accountability Act. A retrospective search was performed using Boolean operators (Folio; Open Market, Proto, UT, USA) to identify all patients who had undergone whole-body FDG-PET at our institution from 2003 to 2006 and had the finding of a compression fracture mentioned in the PET report. Imaging studies and reports, medical records, and pathology reports of selected cases were reviewed.

### Patients

We identified 33 patients with compression fractures who underwent whole-body FDG-PET. There were 23 women and 10 men, aged 48–93 years, with a mean age of 72 years. Twenty-nine patients had a history of malignancy (1 had leukemia, 5 lung cancer, 1 ovarian cancer, 2 breast cancer , 5 colon cancer, 9 lymphoma, 1 sarcoma, 1 pancreatic cancer, 1 Klatskin tumor, 1 gastrointestinal stromal tumor, 1 laryngeal cancer, and 1 had esophageal cancer). The 4 patients without history of malignancy underwent whole body PET for work-up of indeterminate lung nodules detected on prior CT or radiographs. Sixteen patients underwent FDG-PET and 17 patients underwent FDG-PET/CT at time of evaluation. Nine patients underwent CT and 14 patients underwent MRI of the spine within 4 weeks of FDG-PET. Seventeen patients were followed up with serial FDG-PET studies. Nine patients underwent biopsy and the pathologic results were used as a standard of reference. Twenty-four patients who did not undergo biopsy were followed up clinically and with repeat imaging, with MRI, CT, or FDG-PET for a period of 1–3 years and findings at clinical follow-up and imaging studies were used for lesion verification. In these patients, the development of new lesions and/or progression of existing lesions on imaging were used as criteria for malignant compression fractures.

### Image acquisition

Whole-body PET was performed using an ECAT HR+ scanner (CTI Molecular Imaging, Knoxville, TN, USA). All patients fasted for at least 6 h prior to image acquisition, and blood glucose levels were measured prior to the injection of FDG. A dose of 15–20 mCi (555–740 MBq) of FDG was administered intravenously 45 min to 1 h prior to scanning.

Patients were positioned supine on the scanner and emission images were acquired in 6–7 bed positions from the mandible to the mid-thigh or to the level of the ankles in the case of lower extremity lesions. Transmission images obtained with a rotating germanium 68-rod source were used for attenuation correction. Images were reconstructed using the ordered-subset expectation maximization (OSEM) algorithm.

Combined PET/CT studies were performed with a 16-section hybrid PET/CT gantry (Biograph Sensation 16; Siemens, Erlangen, Germany), which comprises a 16-section high-performance multi-detector row CT scanner with a lutetium oxyorthosilicate-based PET scanner. The PET image spatial resolution was 5.0 mm full width at half maximum, with a section thickness of 3.5 mm. Patients fasted for at least 6 h prior to image acquisition, and blood glucose levels were measured prior to the injection of FDG. Two 10-oz cups of water were administered as negative contrast material 1 h prior to scanning. A dose of 15–20 mCi (555–740 MBq) of FDG was administered intravenously 45 min to 1 h prior to scanning. Patients were positioned supine on the scanner and emission images were acquired in 6–7 bed positions from the mandible to mid-thigh or to the level of the ankles in the case of lower extremity lesions. Images were reconstructed with Fourier rebinning and attenuation-weighted ordered-subsets expectation maximization. A low-dose CT scan was performed prior to PET imaging primarily for attenuation correction, with patients holding their breath mid-expiration, and included an area from the mandible to the mid-thigh or to the level of the ankles in the case of lower extremity lesions. Slice thickness was 5 mm. A diagnostic contrast-enhanced CT was performed subsequent to the PET/CT following the administration of 100 mL intravenous contrast material (Isovue 300; Bracco Diagnostics, Princeton, NJ, USA) at an injection rate of 2 mL/s using 2.5-mm sections.

### Image analysis

Semiquantitative and qualitative evaluation of PET images was performed on a high-resolution workstation (Reveal-MVS; Mirada Solutions, Oxford, UK) by one investigator, who was blinded to the clinical and pathological results (MAB). The images were displayed in rotating maximum intensity projections and in axial, coronal, and sagittal planes. Semiquantitative analysis of FDG uptake was performed by creating a region of interest over the area of maximal radiotracer activity.

Maximum standardized uptake values (SUVs) were automatically generated according to the following equation: SUV_max (bw)_ = C_tis_/D_inj_/bw, where SUV_max (bw)_ is maximum SUV normalized for body weight; C_tis_, tissue concentration expressed as megabecquerels per milliliter; D_inj_, injected dose expressed as megabecquerels; and bw, body weight expressed as kilograms. Lesions with SUV greater than 3.0 were considered malignant and less then 3.0 were considered benign. However, lesions with SUV between 2 and 3 are often indeterminate and do not always allow definite diagnosis, and also the pattern of uptake has to be considered. Qualitative assessment was made with the specific aim of establishing whether the lesion was benign or malignant. The radiotracer uptake by the lesion was compared with the liver, and those lesions with uptake greater than the liver were classified as malignant.

### Statistical analysis

The recorded data were analyzed using JMP statistical database software (SAS Institute, Cary, NC, USA). The findings on the PET images as well as SUV of benign and malignant compression fractures were correlated with the final diagnosis of the lesion and determined either malignant or benign. Sensitivity, specificity, positive predictive value, negative predictive value, and accuracy were calculated. The Student’s *t* test was used to determine whether there was a statistically significant difference between the SUV for benign and malignant compression fractures. A difference with *p* < 0.05 was considered to be statistically significant.

## Results

Forty-three compression fractures were identified in 33 patients. Twenty-two fractures involved the thoracic and 21 fractures the lumbar spine. Nine patients underwent biopsy and 24 patients were followed up clinically and with repeat imaging. In the 9 patients who underwent biopsy, there were 3 benign and 6 malignant compression fractures. Of the 24 patients who were followed clinically and with repeat imaging, 6 patients were thought to have malignant and 18 patients were thought to have benign compression fractures. Overall, there were 21 patients with benign and 12 patients with malignant compression fractures. Out of the 43 compression fractures, there were 29 benign and 14 malignant fractures.

Based on clinical history (acute onset of back pain often after minor trauma) and imaging characteristics on MRI (bone marrow edema) there were 5 acute benign compression fractures. Three patients were on bone marrow-stimulating therapy at the time of FDG-PET. Five patients were unable to undergo MRI because of pacemakers (3) or severe pain.

### Qualitative PET analysis

**Fig. 1:**
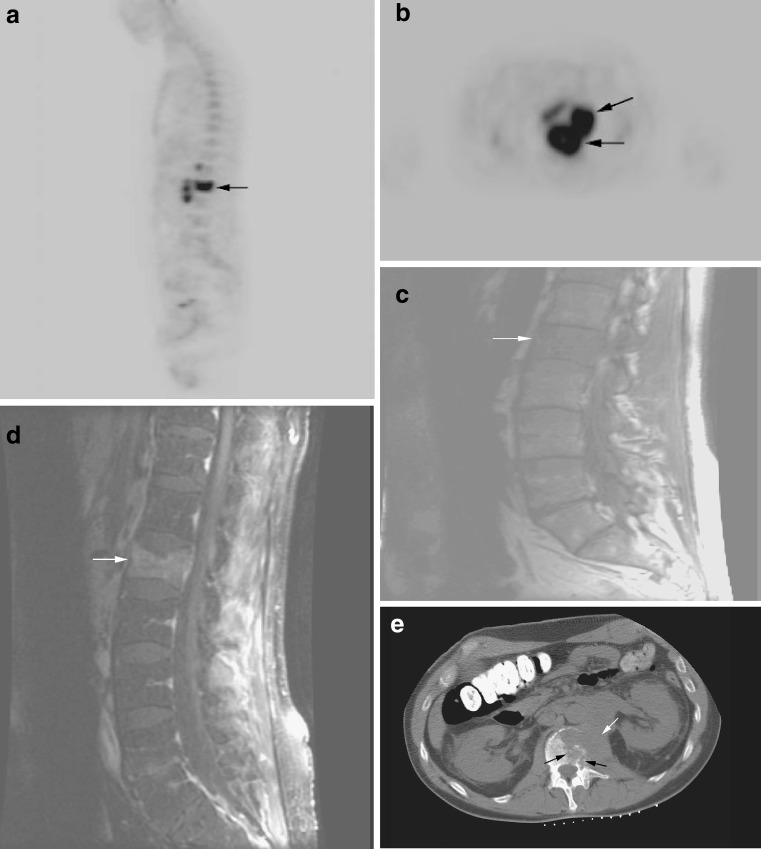
**a** Malignant compression fracture in a patient with lymphoma. Sagittal FDG-PET image demonstrates intense radiotracer uptake in the L1 vertebral body (SUV = 7.1;* arrow*). **b** Axial FDG-PET image demonstrates tumor infiltration of the L1 vertebral body with associated extra-osseous component (*arrows*). **c** Sagittal T1-weighted MRI demonstrates diffuse low signal intensity of the bone marrow of the lumbar spine with compression fracture of L1 (*arrow*). **d** Sagittal fat-suppressed T1-weighted MRI following the administration of gadolinium demonstrates diffuse enhancement of the L1 compression fracture (*arrow*). **e** Axial image from a CT-guided biopsy demonstrates large extra-osseous soft tissue component (*white arrow*) and infiltration of the L1 vertebral body (*black arrows*)

**Fig. 2:**
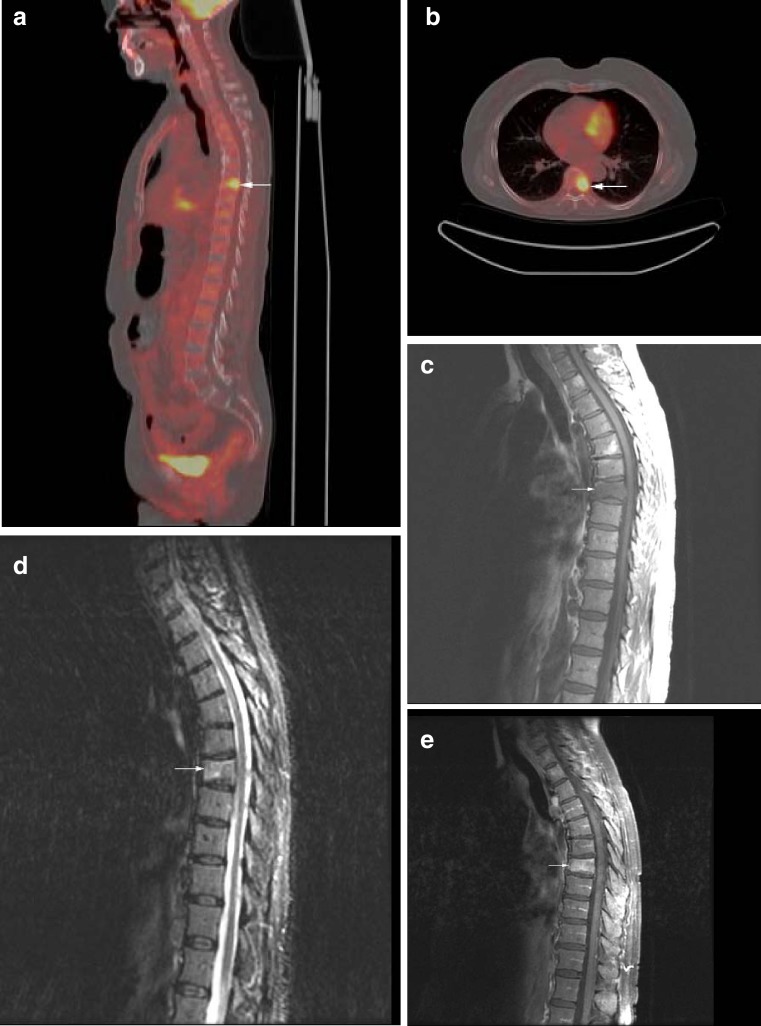
**a** Malignant compression fracture from metastatic colon cancer. Sagittal fused FDG-PET/CT image demonstrates intense radiotracer uptake in the T7 compression fracture (SUV = 6.3;* arrow*). **b** Axial fused FDG-PET/CT image demonstrates focal increased radiotracer uptake in the T7 vertebral body (*arrow*). **c** Sagittal T1-weighted MRI demonstrates compression fracture of T7 with low signal marrow infiltration (*arrow*). **d** Sagittal STIR image demonstrates mild hyperintensity of the T7 vertebral compression fracture (*arrow*). **e** Sagittal fat-suppressed T1-weighted MRI following the administration of gadolinium demonstrates diffuse enhancement of the T7 compression fracture (*arrow*). Malignancy was indeterminate on MRI

**Fig. 3:**
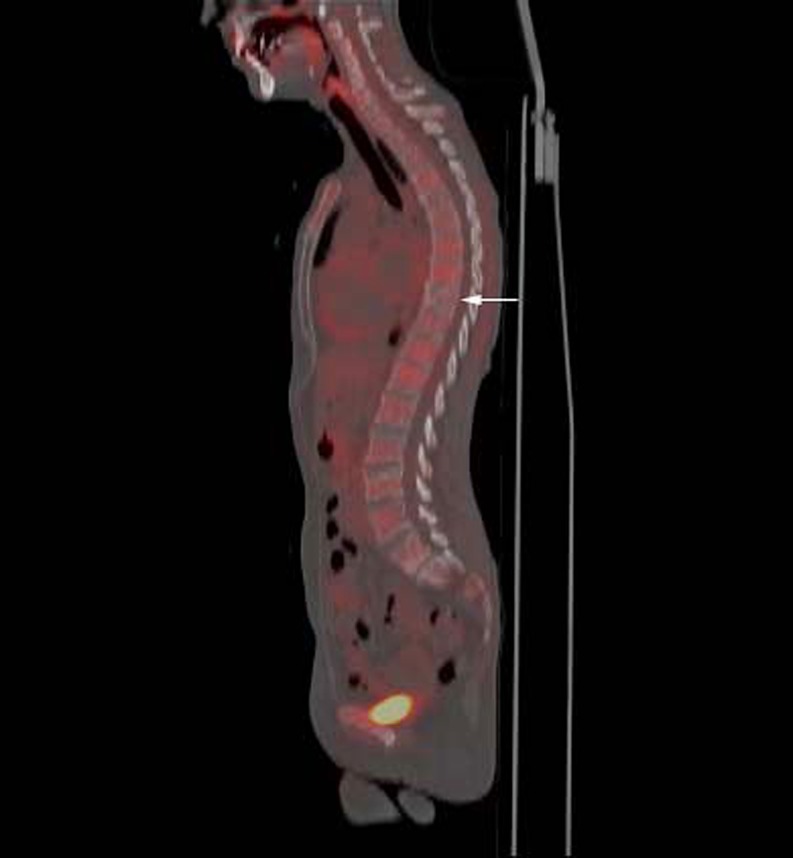
Benign compression fracture in a patient with lymphoma. Sagittal fused FDG-PET/CT image demonstrates compression fracture of T9 (*arrow*) without significant radiotracer uptake (SUV = 0.7)

**Fig. 4:**
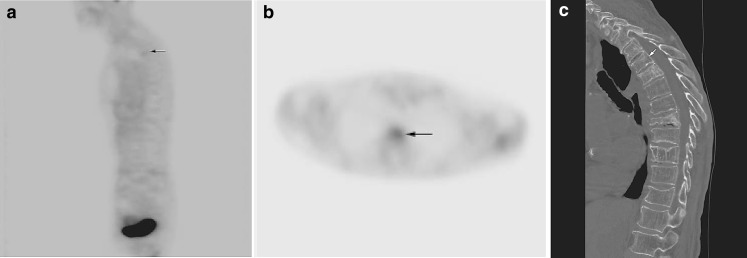
**a** Benign subacute compression fractures in a patient without history of malignancy. Sagittal FDG-PET image demonstrates mildly increased radiotracer uptake in the upper thoracic spine (SUV = 1.8; *arrow*). **b** Axial FDG-PET image at the level of T4 demonstrates mildly increased radiotracer uptake (*arrow*), consistent with subacute compression fracture. **c** Sagittal CT image demonstrates compression fracture of T4 (*arrow*) and multiple chronic osteoporotic compression fractures throughout the thoracic spine

**Fig. 5:**
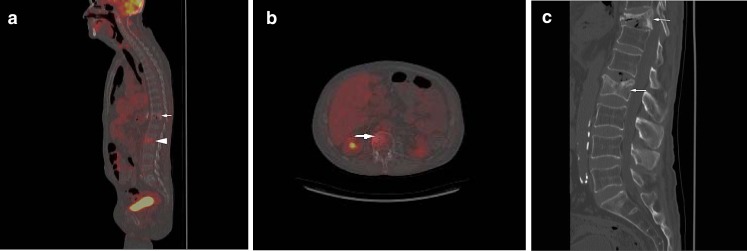
**a** False-negative compression fracture on FDG-PET in a patient with metastatic esophageal cancer. Sagittal fused FDG-PET/CT image demonstrates mild to moderately increased radiotracer uptake in the L2 compression fracture (*arrow*; SUV = 2.5) that was thought to represent a benign compression fracture but was found to be malignant on subsequent biopsy. Note the mildly increased radiotracer uptake in the T11 compression fracture (*arrowhead*), which was thought to represent a benign fracture. **b** Axial fused FDG-PET/CT image demonstrates increased radiotracer uptake in the L2 vertebral body (*arrow*). **c** Sagittal CT image demonstrates L2 and T11 compression fractures (*arrows*), for which malignancy was indeterminate

Forty-three compression fractures were identified in 33 patients. Visual inspection of suspected lesions on FDG-PET characterized 26 compression fractures as benign and 17 fractures as malignant. The malignant lesions demonstrated intense radiotracer uptake (Figs. 1, 2), while benign compression fractures showed only mildly increased or no increased uptake on FDG-PET (Figs. 3, 4). Histological and clinical follow-up data were analyzed. By FDG-PET, 12 lesions were correctly classified as malignant (true-positive) and 24 lesions were correctly classified as benign (true-negative). Two malignant tumors were incorrectly classified as benign (false-negative) and 5 benign tumors were incorrectly classified as malignant (false-positive). In the 2 false-negative cases there was moderately increased uptake of the compression fractures, which was thought to represent acute benign fractures prospectively, but was found to be metastatic disease on subsequent biopsy (Fig. 5). SUV in these patients were 2.5 to 2.8 respectively, and the primaries in these cases were esophageal and lung cancer.

**Fig. 6:**
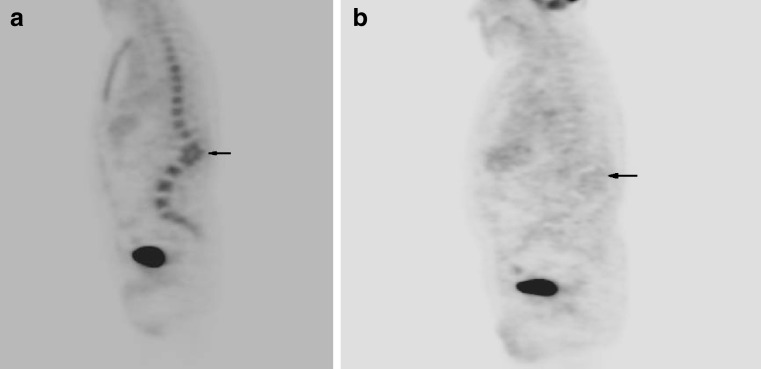
**a** False-positive compression fracture in a patient on bone marrow-stimulating agents. Sagittal FDG-PET image demonstrates diffuse increased radiotracer activity throughout the spine. Note the compression fractures with focal kyphotic angulation at L1–L2 (*arrow*). The patient was thought to have diffuse metastatic disease with malignant compression fracture. **b** Sagittal FDG-PET image performed 2 months later demonstrates resolution of diffuse radiotracer uptake. Kyphotic angulation from compression fractures remains (*arrow*). The patient was off bone marrow-stimulating agents for 6 weeks at the time of the study

**Fig. 7:**
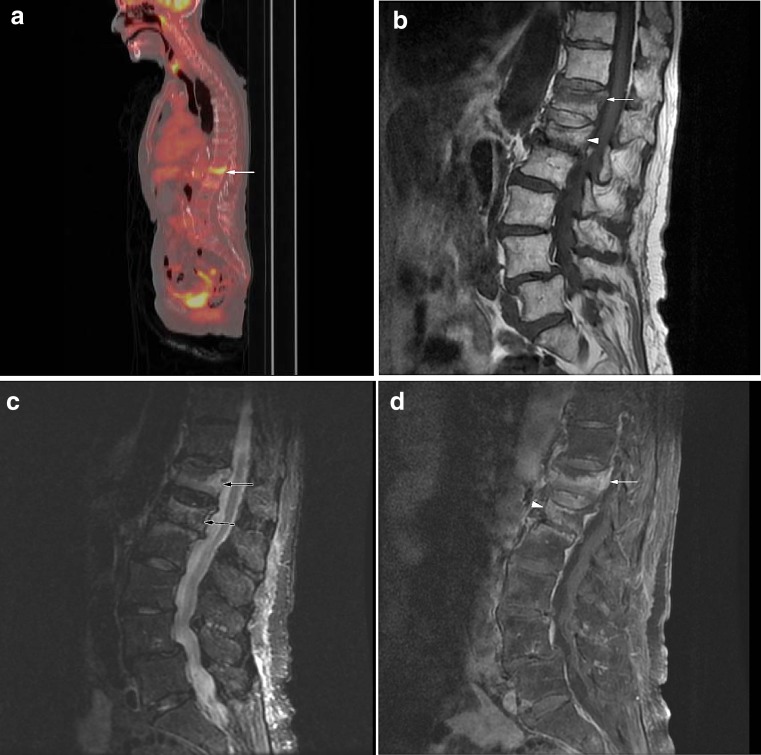
**a** False-positive compression fracture in a patient with laryngeal cancer. Sagittal fused FDG-PET/CT image demonstrates increased radiotracer uptake in the T12 compression fracture (SUV = 4.9;* arrow*). **b** Sagittal T1-weighted MRI demonstrates compression fracture of T12 with linear low signal of the superior endplate and preservation of the normal marrow signal (*arrow*). Note compression fracture of L1 with relative preservation of normal marrow signal (*arrowhead*). **c** Sagittal STIR image demonstrates hyperintensity of the superior endplates of the T12 and L1 compression fractures, suggesting acute fractures (*arrows*). **d** Sagittal fat-suppressed T1-weighted MRI following the administration of gadolinium demonstrates enhancement of the T12 compression fracture (*arrow*). Mild enhancement is noted, involving the L1 compression fracture (*arrowhead*). Findings were thought to be benign on MRI and on follow-up imaging, there was resolution of bone marrow edema and enhancement

**Fig. 8:**
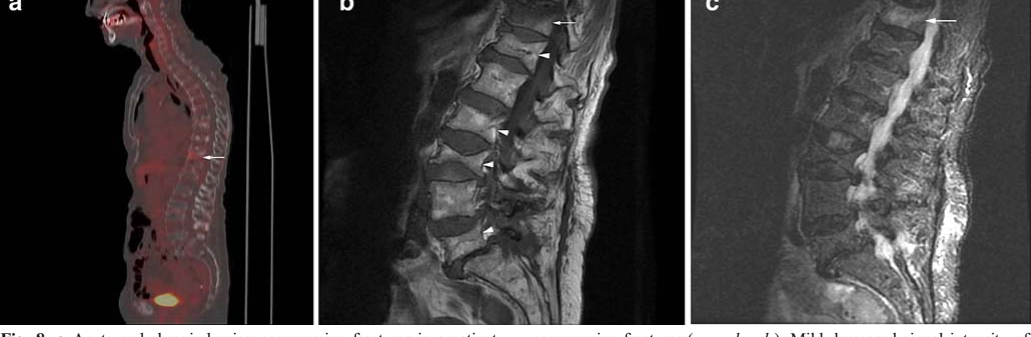
**a** Acute and chronic benign compression fractures in a patient without history of malignancy. Sagittal fused FDG-PET/CT image demonstrates multiple compression fractures throughout the thoracic and lumbar spine with mild increased radiotracer uptake at T11 (SUV = 2.1;* arrow*). **b** Sagittal T1-weighted MRI demonstrates multiple chronic compression fractures (*arrowheads*). Mild decreased signal intensity of the T11 compression fracture suggests edema (*arrow*). **c** Sagittal STIR image demonstrates mild edema in the T11 compression fracture suggestive of an acute fracture (*arrow*). There is no evidence of marrow edema in the remaining compression fractures

Three of the false-positive patients were on bone marrow-stimulating agents (Fig. 6). One of the false-positive patients had an acute compression fracture (Fig. 7). Except for that patient, there was no significant difference in uptake pattern of benign acute and benign chronic compression fractures (Fig. 8).

### Quantitative PET analysis

**Fig. 9:**
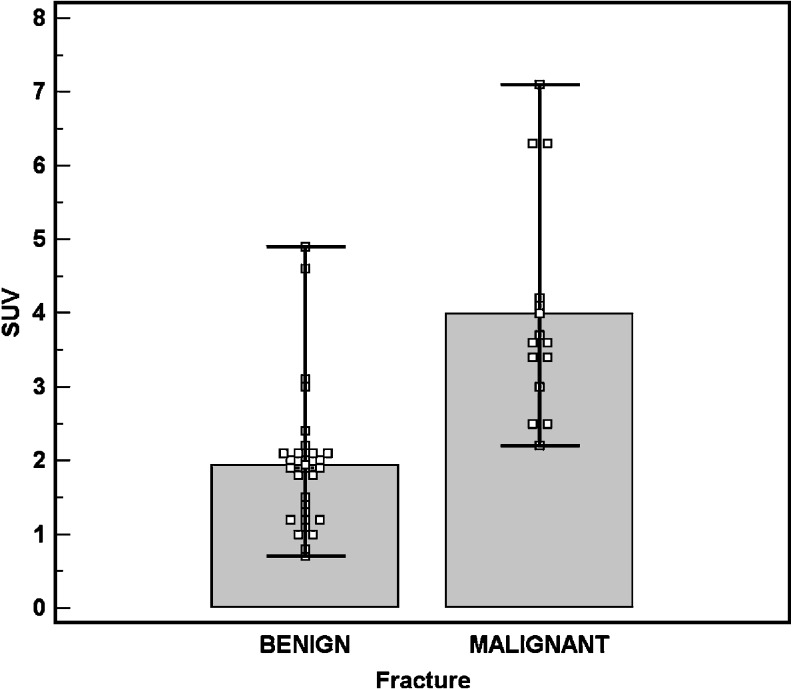
Mean standardized uptake value (SUV) of benign and malignant compression fractures. The top of the boxes represent the mean and error bars represent the range of SUV. There is a statistically significant difference between the SUV values of benign and those of malignant compression fractures (*p* < 0.001, Student’s *t* test)

Standardized uptake values were measured in all 43 compression fractures. SUV for benign compression fractures ranged from 0.7 to 4.9 with a mean of 1.94 ± 0.97 standard deviation (SD) on FDG-PET. Malignant compression fractures showed SUV from 2.2 to 7.1 with a mean of 3.99 ± 1.52 SD on FDG-PET. The difference between the SUV values of benign and malignant compression fractures was statistically significant (*p* < 0.001, Student’s *t* test; Fig. 9).

### Statistical analysis

Sensitivity, specificity, positive and negative predictive values, and accuracy of FDG-PET in differentiating benign from malignant compression fractures were 86%, 83%, 84%, 71%, and 92% respectively.

## Discussion

The correct diagnosis of benign and malignant compression fractures can be problematic, but has important prognostic and therapeutic implications. MRI, CT, or bone scintigraphy are commonly used in the diagnostic work-up of patients with compression fractures. However, in some cases, these imaging techniques do not permit definite diagnosis of the cause of the compression fracture. Diffusion-weighted MRI has been used successfully to differentiate benign from malignant fractures [10–13], but benign and malignant compression fractures can show significant overlap on quantitative assessment with apparent diffusion coefficient maps [12]. Also, some patients are unable to undergo MRI due to pacemakers, claustrophobia, or severe pain. This is especially true in the elderly population. Five of our 33 patients were unable to undergo MRI.

In this context, metabolic imaging modalities such as FDG-PET might be used as a management problem solver. FDG-PET has been successfully used to differentiate benign from malignant neoplasms and in the evaluation of metastatic disease [14–16]. Preliminary studies and case reports have shown that FDG-PET might be helpful in differentiating benign from malignant compression fractures [8, 9]. Since elderly patients often have a history of malignancy and are also predisposed to benign compression fractures due to osteoporosis, we thought that FDG-PET might be used as a diagnostic problem solver in this patient population in cases of equivocal MRI or CT findings or if the patient was unable to undergo MRI.

In our study, malignant compression fractures demonstrated significantly increased FDG uptake compared with benign fractures. Mean SUV of malignant and benign fractures were 3.99 ± 1.52 SD for malignant lesions and 1.94 ± 0.97 SD for benign lesions. There were 2 false-negative results. In these cases there was moderately increased uptake of the compression fractures, which were thought to be acute benign fractures prospectively, but were found to represent metastatic disease on subsequent biopsy. SUV in these patients were 2.5 to 2.8 respectively.

There were 5 false-positive results; 3 of those patients were on bone marrow-stimulating agents, which mimicked tumor involvement. This effect on bone marrow FDG uptake has been described in the literature [17]. One to 2 months after cessation of bone marrow-stimulating therapy, FDG uptake returned to normal in our patients. The false-positive results occurred because the investigator was blinded to the clinical history. This is less likely to occur in a clinical setting where this history should be actively sought when interpreting positive findings, especially in cases of diffuse osseous uptake, mimicking a diffuse marrow infiltrative process as was seen in our cases. In fact, when we reviewed the original reports, these cases were reported to be consistent with the known use of bone marrow-stimulating agents.

Only mildly increased or no increased uptake was seen in chronic benign fractures and mild to moderately increased uptake was seen in acute benign fractures, which was less than in malignant fractures. This is in contrast to bone scintigraphy, in which increased uptake persists for many months [18].

Positron emission tomography and PET/CT were equally sensitive in differentiating benign from malignant compression fractures. However, the CT portion of the PET/CT improved the exact fracture localization and was able to provide additional information on fracture morphology, which can be helpful in diagnosing benign vs. malignant fractures.

Our study had several limitations. The first is the retrospective nature of the study. Second is the lack of histologic correlation in all cases. Only 9 patients underwent biopsy of the spine. However, we obtained clinical follow-up and serial imaging studies, including serial FDG-PET for a period of 1–3 years to evaluate for benign or malignant fractures. Also, there were no patients with osteomyelitis/discitis, which can mimic malignancy [8, 19, 20]. Another limitation is the lack of inter-observer variability, since only one observer interpreted the images. However, we compared our results with the reports of the original interpreter, who had access to all data. On the original reports, the 3 cases of patients on bone marrow-stimulating agents were interpreted as being consistent with the known use of bone marrow-stimulating agents. Malignancy was said to be indeterminate in the two false-negative reports. However, in our review, we categorized each fracture as either benign or malignant without the option of an indeterminate lesion.

In summary, FDG-PET is a useful method of differentiating between benign and malignant compression fractures and can serve as a problem solver in cases of equivocal MRI or CT findings, and in patients who are unable to undergo MRI. We do not recommend FDG-PET as a screening test, but rather as an additional imaging modality in problem cases, particularly in elderly patients with osteoporosis and a history of malignancy. In these patients, FDG-PET has the additional advantage of being able to evaluate the entire skeletal system and screen for metastatic disease.

## References

[1] Fayad LM, Kamel IR, Kawamoto S, Bluemke DA, Frassica FJ, Fishman EK (2005). Distinguishing stress fractures from pathologic fractures: a multimodality approach. Skeletal Radiol.

[2] Kubota T, Yamada K, Ito H, Kizu O, Nishimura T (2005). High-resolution imaging of the spine using multidetector-row computed tomography: differentiation between benign and malignant vertebral compression fractures. J Comput Assist Tomogr.

[3] Uetani M, Hashmi R, Hayashi K (2004). Malignant and benign compression fractures: differentiation and diagnostic pitfalls on MRI. Clin Radiol.

[4] Ho CS, Choi WM, Chen CY, Chen WY, Chan WP (2005). Metastasis in vertebra mimicking acute compression fractures in a patient with osteoporosis: MRI findings. Clin Imaging.

[5] Li KC, Poon PY (1988). Sensitivity and specificity of MRI in detecting malignant spinal cord compression and in distinguishing malignant from benign compression fractures of vertebrae. Magn Reson Imaging.

[6] Dehdashti F, Siegel BE, Griffeth LK (1996). Benign versus malignant intraosseous lesions: discrimination by means of PET with 2-[F-18]fluoro-2-deoxy-D-glucose. Radiology.

[7] Glaspy JA, Hawkins R, Hoh CK, Phelps ME (1993). Use of positron emission tomography in oncology. Oncology (Williston Park).

[8] Schmitz A, Risse JH, Textor J (2002). FDG-PET findings of vertebral compression fractures in osteoporosis: preliminary results. Osteoporos Int.

[9] Shon IH, Fogelman I (2003). F-18 FDG positron emission tomography and benign fractures. Clin Nucl Med.

[10] Chan JH, Peh WC, Tsui EY (2002). Acute vertebral body compression fractures: discrimination between benign and malignant causes using apparent diffusion coefficients. Br J Radiol.

[11] Herneth AM, Phillip MO, Naude J (2002). Vertebral metastases: assessment with apparent diffusion coefficient. Radiology.

[12] Maeda M, Sakuma H, Maier SE, Takeda K (2003). Quantitative assessment of diffusion abnormalities in benign and malignant vertebral compression fractures by line scan diffusion-weighted imaging. AJR Am J Roentgenol.

[13] Raya JG, Dietrich O, Reiser MF, Baur-Melnyk A (2006). Methods and applications of diffusion imaging of vertebral bone marrow. J Magn Reson Imaging.

[14] Adler LP, Blair HF, Makley JT (1991). Noninvasive grading of musculoskeletal tumors using PET. J Nucl Med.

[15] Aoki J, Watanabe H, Shinozaki T (2001). FDG PET of primary benign and malignant bone tumors: standardized uptake value in 52 lesions. Radiology.

[16] Franzius C, Sciuk J, Jürgens HE, Schober J (2000). FDG-PET for detection of osseous metastases from malignant primary bone tumours: comparison with bone scintigraphy. Eur J Nucl Med.

[17] Kazama T, Swanston N, Podoloff DA, Macapinlac H (2005). Effect of colony-stimulating factor and conventional- or high-dose chemotherapy on FDG uptake in bone marrow. Eur J Nucl Med Mol Imaging.

[18] Masala S, Schillaci O, Massari F (2005). MRI and bone scan imaging in the preoperative evaluation of painful vertebral fractures treated with vertebroplasty and kyphoplasty. In Vivo.

[19] Guhlmann A, Brecht-Krauss D, Suger G (1998). Fluorine-18-FDG PET and technetium-99m antigranulocyte antibody scintigraphy in chronic osteomyelitis. J Nucl Med.

[20] Guhlmann A, Brecht-Krauss D, Suger G (1998). Chronic osteomyelitis: detection with FDG PET and correlation with histopathologic findings. Radiology.

